# The effectiveness of a preferred intensity exercise programme on the mental health outcomes of young people with depression: a sequential mixed methods evaluation

**DOI:** 10.1186/1471-2458-12-187

**Published:** 2012-03-13

**Authors:** Tim Carter, Patrick Callaghan, Elizabeth Khalil, Ioannis Morres

**Affiliations:** 1 School of Nursing, Midwifery & Physiotherapy, University of Nottingham, Nottingham, UK

**Keywords:** Depression, Adolescence, Young people, Exercise, Physical activity

## Abstract

**Background:**

People with mental illness are more likely to suffer physical health problems than comparable populations who do not have mental illness. There is evidence to suggest that exercise, as well has having obvious physical benefits, also has positive effects on mental health. There is a distinct paucity of research testing its effects on young people seeking help for mental health issues. Additionally, it is generally found that compliance with prescribed exercise programmes is low. As such, encouraging young people to exercise at levels recommended by national guidelines may be unrealistic considering their struggle with mental health difficulties. It is proposed that an exercise intervention tailored to young people's preferred intensity may improve mental health outcomes, overall quality of life, and reduce exercise attrition rates.

**Methods/Design:**

A sequential mixed methods design will be utilised to assess the effectiveness of an individually tailored exercise programme on the mental health outcomes of young people with depression. The mixed methods design incorporates a Randomised Controlled Trial (RCT), focus groups and interviews and an economic evaluation. *Participants: *158 young people (14-17 years) recruited from primary care and voluntary services randomly allocated to either the intervention group or control group. *Intervention group*: Participants will undertake a 12 week exercise programme of 12 × 60 minutes of preferred intensity aerobic exercise receiving motivational coaching and support throughout. Participants will also be invited to attend focus groups and 1-1 interviews following completion of the exercise programme to illicit potential barriers facilitators to participation. *Control group*: Participants will receive treatment as usual. *Primary Outcome measure*: Depression using the Children's Depression Inventory 2 (CDI-2). *Secondary Outcome measures*: Quality of Life (EQ-5D), physical fitness (Borg RPE scale, heart rate), incidents of self-harm, treatment received and compliance with treatment, and the cost effectiveness of the intervention. Outcome measures will be taken at baseline, post intervention and 6 month follow up.

**Discussion:**

The results of this study will inform policy makers of the effectiveness of preferred intensity exercise on the mental health outcomes of young people with depression, the acceptability of such an intervention to this population and its cost effectiveness.

**Trial Registration:**

ClinicalTrials.gov: NCT01474837

## Background

The Department of Health in England has identified that people who use mental health services, in particular those with diagnosis of schizophrenia or bipolar disorder, are at increased risk of coronary heart disease, diabetes, infections, respiratory disease and greater levels of obesity [1]. Additionally, it is reported that they are almost twice as likely to die from coronary heart disease and up to four times as likely to die from respiratory disorder as those in the general population [2,3]. Consequently, the Department of Health launched a national initiative to improve the physical health of people with mental illness with an aim to promote activity for all [4].

The numerous physical benefits of exercise have been well evidenced throughout the years and are abundant in health promotion literature in G.P surgerys, leisure centres and gymnasiums. Engaging in exercise is associated with reducing the risk of developing heart disease, stroke, cancer, type 2 diabetes and obesity and promoting musculoskeletal health [4]. It improves the poor physical health that leads to morbidity and early deaths in mentally ill populations.

There is a breadth of evidence suggesting that exercise also has positive effects on mental health. Generally it has been found that exercise can have a positive impact on mood, self esteem and self worth [5]. Importantly, it has also been shown to be effective at reducing symptoms of depression and anxiety, this has been evidenced in both the general population [6-8] and in adult clinical populations [9,10]. Although the evidence is scarce there are some studies investigating the association of exercise on mental health, in particular on depression in young people.

Drawing on evidence from a national school based study looking into depression and suicidal ideation of adolescents it was found that higher levels of depression were associated with lower levels of sport participation. After controlling for covariates it was reported that participation in sports reduced the odds of suffering from depression by 25% [11]. Similar reports have been found from longitudinal data; in a 6 Year study of 496 adolescent students, in which assessments of physical activity and depression scores were taken at 1-year intervals, baseline physical activity predicted fewer depressive symptoms over time [12]. This finding is supported by longitudinal studies [13,14].

Experimental data is scarce in the literature and results are mixed; In an RCT comparing participants receiving 24 sessions of aerobic activities of moderate intensity compared to a light contact placebo group and a non-treatment control group, participants in the intervention group improved on measures of self-esteem relative to the control group, however no differences were shown on depression scores [15]. Conversely, in an RCT in which 66 Hispanic students were randomised to an intervention group consisting of moderate intensity aerobic exercise or a control group of low intensity physical activity over a 6 week period, depression scores were significantly lower post intervention in the intervention group [16].

Although there are a wealth of studies testing the association between exercise and mental health in young people in the general population, there is little data on its association in clinical populations, i.e. those seeking treatment for mental health issues. In a systematic review of clinical trials up to 2005 [17] only three trials were found to have investigated the effect of exercise on this population [18-20]. All three studies reported no significant difference between depression scores in the intervention and control groups. However, two of the studies were reported to be of low quality and one was rated as moderate; all three studies had relatively small sample sizes. More recently there have been two studies investigating the effects of exercise on young people in a clinical population with slightly more positive results. In a single measures design study, 15 female participants meeting the Diagnostic & Statistical Manual for Mental Disorders IV (DSM-IV) criteria for Post-Traumatic Stress Disorder (PTSD) undertook an aerobic exercise programme for 40 minutes 3 times a week for12 weeks. Significant reduction in depression scores were reported from baseline to mid intervention and post intervention. However, a significant increase in depression scores was reported at 1 month follow up when comparing post intervention to 1 month. The authors conclude that aerobic training may be an effective intervention for childhood PTSD symptom reduction. However, increase in depression scores at 1 month follow up suggests that reduction in depression is not maintained when exercise ceases [21]. Another single case repeated measures study using 12 female participants (aged 14-17) receiving treatment from a PTSD treatment centre in which participants undertook 25 minute sessions of moderate intensity aerobic exercise 3 times a week for 5 weeks reported that 2 of the 8 participants who demonstrated stable depression scores at baseline showed significant reduction in depression scores post intervention. Importantly, both studies had inadequate sample sizes and no control group and one had a large variance in the type of physical activity participants undertook.

In summary, it is clear that there is potential for exercise to have an effect on depression scores in young adults with depression, however to the authors' best knowledge there has been no well designed RCT's with sufficient statistical power investigating this issue. Furthermore, it is felt that encouraging young people to exercise at levels recommended by national guidelines, or as prescribed by the researcher may be unrealistic for young people with mental health difficulties. The researchers' previous work among young people suggests that interventions tailored to young people's preferred intensity may improve their motivation for exercise and reduce attrition rates and lead to improved mental health outcomes, increase self-esteem and overall quality of life. Therefore, a specially designed programme with exercise at a preferred intensity has the potential to improve health outcomes in young people.

### Objectives

1. To determine the effectiveness of a preferred intensity exercise programme on the mental health outcomes of young people with depression.

2. To examine the acceptability of a preferred intensity exercise programme to young people with depression

3. To determine the cost effectiveness of a preferred intensity exercise programme for young people with depression.

### Hypothesis

It is hypothesised that exercise of preferred intensity will significantly lower depression scores, increase quality of life, reduce incidents of self harm and be more cost-effective than treatment as usual.

## Method/Design

A sequential mixed methods design will be utilised and consists of 3 parts: A Randomised Controlled Trial (RCT), focus groups and interviews, and an economic evaluation. The RCT: Participants will be randomly allocated to preferred intensity aerobic exercise (intervention arm) or treatment as usual (control arm). Outcome measures, which will be collected independently of the research team, will be captured at baseline, post-intervention and six months follow-up. Qualitative data will be collected through face-to-face qualitative interviews and focus groups to understand potential barriers or facilitators to participation. An economic evaluation will also be conducted in order to assess the cost effectiveness of the intervention.

### Sample & recruitment

Participants will be young people aged 14-17 recruited from Primary Care Services, Child and Adolescent Mental Health Services and Voluntary Services in Nottingham City and County in the UK.

The initial approach will be from a member of the patient's usual care team who will be informed about the study. The participants then will be referred by his/her GP/Dr/Health Worker who believes that he/she may benefit from this programme because he/she have been having some difficulties with his/her mental health.

### Inclusion/exclusion criteria

Inclusion Criteria - Young person to be aged between 14-17, receiving treatment for depression or scoring as depressed on the Children's Depression Inventory (CDI)

Exclusion Criteria- participants having any injury or physical health problem or condition that precludes their participation.

### Screening

Screening will take place by the potential participant's GP or Mental Health Professional who will be given a flow chart of inclusion criteria. Alternatively, if the participant has been referred by non mental health community/voluntary services he/she will be screened by a member of the research team who will administer the Children's Depression Inventory (CDI).

### Primary outcome measure

#### The Children's Depression Inventory 2(CDI-2) [22]

The CDI-2 consists of 28-items designed to assess depression scores in young people aged 7 to 17. For each item the there are three possible answers; 0 indicating the absence of symptoms, 1 the mild symptoms, and 2 the definite symptoms. The total score ranges from 0 to 54. The CDI is sensitive to changes in depressive symptoms over time and is a useful index of the severity of the depressive syndrome.

### Secondary outcome measures

#### European Quality of Life-5 Dimensions (EQ-5D)

The EQ-5D is a standardised measure of health-related quality of life that provides a simple, generic measure of health for clinical and economic appraisal^. ^It comprises five questions on mobility, self care, pain, usual activities, and psychological status with five possible answers for each item (1 = no problem, 2 = slight problem, 3 = moderate problem, 4 = severe problem, 5 = unable. It is applicable to a wide range of health conditions and treatments; it provides a simple descriptive profile and a single index value for health status.

#### The Client Receipt of Service Inventory (CRSI)

The CSRI is a questionnaire that collects retrospective information on service utilisation, service-related issues and income in a manner commensurate with estimating care package costs.

#### The Borg (RPE) Scale [23]

THE RPE scale is a simple method of rating perceived exertion (RPE) and to gauge a person's level of intensity when exercising. It has an ordinal scale that ranges from 0-16 with verbal anchors allowing the participants to designate their level of intensity more accurately and for answers to be standardized across individuals and tasks.

Heart Rate

Resting Heart Rate

Self Harm

The number of incidents of self harm in the last 3 months

Compliance with intervention

The number of exercise session each participant attends

### Procedure

The intervention group will undertake 12 × 60 minute aerobic exercise sessions, in groups of 5-10, tailored to participants' exercise levels and preferences led by a qualified exercise therapist. The intervention group will also receive psychosocial and motivational support throughout each session to facilitate ongoing participation and enduring change. The motivational coaching will consist of behaviour modification: goal setting, self-monitoring, social support, enhancing self-efficacy and shaping.

Participants will have baseline measures taken at the initial session. Outcome measures will be repeated at post intervention in the final session and at 6 month follow up at a location that suits the participant.

Participants in the intervention group will be invited to participate in focus groups and face-to-face interviews following completion of the exercise programme. These will take place at a location agreed with participants and researchers. It is hoped that these will be conducted at the centres at which the exercise takes place. The data will be recorded on an audio tape with the informed consent from the participants and their legal guardians. The facilitator will also take written notes which will help identify the participants in the recording. Data will be transcribed, coded and analysed for patterns, themes and sub-categories, paying particular attention to outliers and rival explanations given by participants.

The control group will receive usual care throughout the 12 week intervention period. Outcome measures will be taken within the same week as the paired intervention group; at baseline, post intervention (12 weeks later) and at 6 month follow up.

Resource data will be collected for an economic evaluation using the Client Receipt of Service Inventory (CSRI) which is a comprehensive inventory of resource use that has been widely used in economic evaluations of mental health interventions [24]. Resource use will be valued using published, and where appropriate patient reported, unit costs. The primary measure for the cost effectiveness analysis will be the SPPA (with sensitivity analysis covering a range of possible outcomes). Assessing health related quality of life using the EQ-5D will enable a cost utility analysis to be undertaken for the trial period. Using the information on costs and benefits an incremental economic analysis comparing the tailored exercise intervention to treatment as usual will be undertaken to estimate mean cost-effectiveness. To test the robustness of results in the face or any uncertainties, and in order to improve the generalisability of results, sensitivity analysis will be conducted. In addition, uncertainty surrounding the cost-effectiveness of the tailored exercise intervention will be presented graphically using Cost-Effectiveness Acceptability Curves [25]. Should the data be skewed then non-parametric bootstrap analysis using the percentile method confidence interval [26] will be undertaken and incremental net benefits estimated.

The project steering group will be used to undertake an implementation analysis of the project's findings in order to understand knowledge transfer from research to clinical practice in the health service. Such an approach is in line with MRC guidance for developing and evaluating complex interventions [27]. The MRC guidance highlights that clinical ineffectiveness may be a result of an implementation failure; as a result, it calls for multi-level, process evaluations that may go beyond RCTs to understand such failure. In the steering group we intend to elicit from members the issues relating to the uptake of the findings from the proposed study and the barriers we may encounter in doing this. Each member of the group will complete a proforma eliciting a Social Network Analysis of their interactions with other service providers to better understand the concerns of providers not immediately associated with the project.

### Sample size and justification

128 participants are required in order to have 80% power to detect a difference in mean change of the primary outcome measure. This assumes equal standard deviations of 16 points and using a pre-specified significance level of *p *< 0.05. Assuming 20% attrition rates, it is proposed to recruit 158 participants to the study (79 intervention arm, 79 control arm).

### Stopping rules and discontinuation

The Trial will be stopped if there are significant adverse events that cannot be remedied, if there is a significant failure to recruit the required sample and if requested by the funder or sponsor.

### Randomisation and blinding

Allocated to experimental or control arm will be by computer generated numbers by a researcher not associated with the study. Measurements of baseline, outcome and follow-up measures will be taken by a researcher not associated with the study. Baseline outcome measures will be taken after random allocation. The exercise therapist and focus group facilitator will only know which participants get the exercise intervention.

### Statistical analysis

SPSS V.18 will be used for the analysis of the results. The two groups will be described in terms of their baseline characteristics. Our primary outcome will be the CDI post intervention. A regression model will be used to compare the two groups in terms of the CDI score post intervention conditional on baseline score. No other confounders will be adjusted for unless there appears to be an imbalance at baseline between the two groups on pre-specified covariates that are considered to influence outcome. For secondary outcomes, similar models will be built all conditional on the relevant baseline score in addition to baseline CDI score. Analyses will be intention-to-treat and conducted on a dataset where codes for the two intervention groups are unlabeled. All estimates of effect sizes and numbers needed to treat will be reported with 95% confidence intervals. One of the potential problems with early studies testing a relatively new intervention with a group in whom it is largely untested is that the results may be skewed in a positive direction. To minimise this potential effect, the statistical analyses will be conducted independently of the research team. Qualitative analysis of the focus groups and interviews will be conducted using NVivo 9 in order to code the information and look for relevant trends and themes within the data.

### Ethics

The study received ethical approval from Nottingham Research Ethics Committee on 18/07/11. REC reference: 11/EM/0157.

## Discussion

The results of this study will show whether preferred intensity exercise has an effect on the mental health outcomes of young people with depression and whether it is acceptable to young people with depression.

## Abbreviations

CDI: Children's depression inventory; CSRI: Client receipt of Service inventory; DSM-IV: Diagnostic & statistical manual for mental disorders IV; EQ-5D: European quality of life-5 dimensions; PTSD: Post-traumatic stress disorder; RCT: Randomised controlled trial; RPE: Rating perceived exertion; SPSS: Statistical package for the social sciences.

## Competing interests

The authors declare that they have no competing interests.

## Authors' contributions

TC drafted the manuscript, co-authored the protocol and contributed to the ethics submission. PC conceived of the study, co-authored the protocol and contributed to the ethics submission. EK co-authored the protocol and contributed to the ethics submission. IM co-authored the protocol. All authors contributed to the design of the study, the study protocol and the writing up of the paper. All authors read and approved the final manuscript.

## Pre-publication history

The pre-publication history for this paper can be accessed here:

http://www.biomedcentral.com/1471-2458/12/187/prepub
